# Correction: Enhanced biofilm and extracellular matrix production by chronic carriage versus acute isolates of *Salmonella* Typhi

**DOI:** 10.1371/journal.ppat.1009512

**Published:** 2021-04-12

**Authors:** The PLOS Pathogens Staff

The fourth author’s name is spelled incorrectly. The correct name is: Gaёtan Thilliez. The correct citation is: Devaraj A, González JF, Eichar B, Thilliez G, Kingsley RA, Baker S, et al. (2021) Enhanced biofilm and extracellular matrix production by chronic carriage versus acute isolates of *Salmonella* Typhi. PLoS Pathog 17(1): e1009209. https://doi.org/10.1371/journal.ppat.1009209
