# Supplementary figures and images for: Enhanced biofilm and extracellular matrix production by chronic carriage versus acute isolates of Salmonella Typhi
Source: PLoS Pathog. 2021 Jan 19;17(1):e1009209. doi: 10.1371/journal.ppat.1009209 (PMC7815147; doi:10.1371/journal.ppat.1009209)

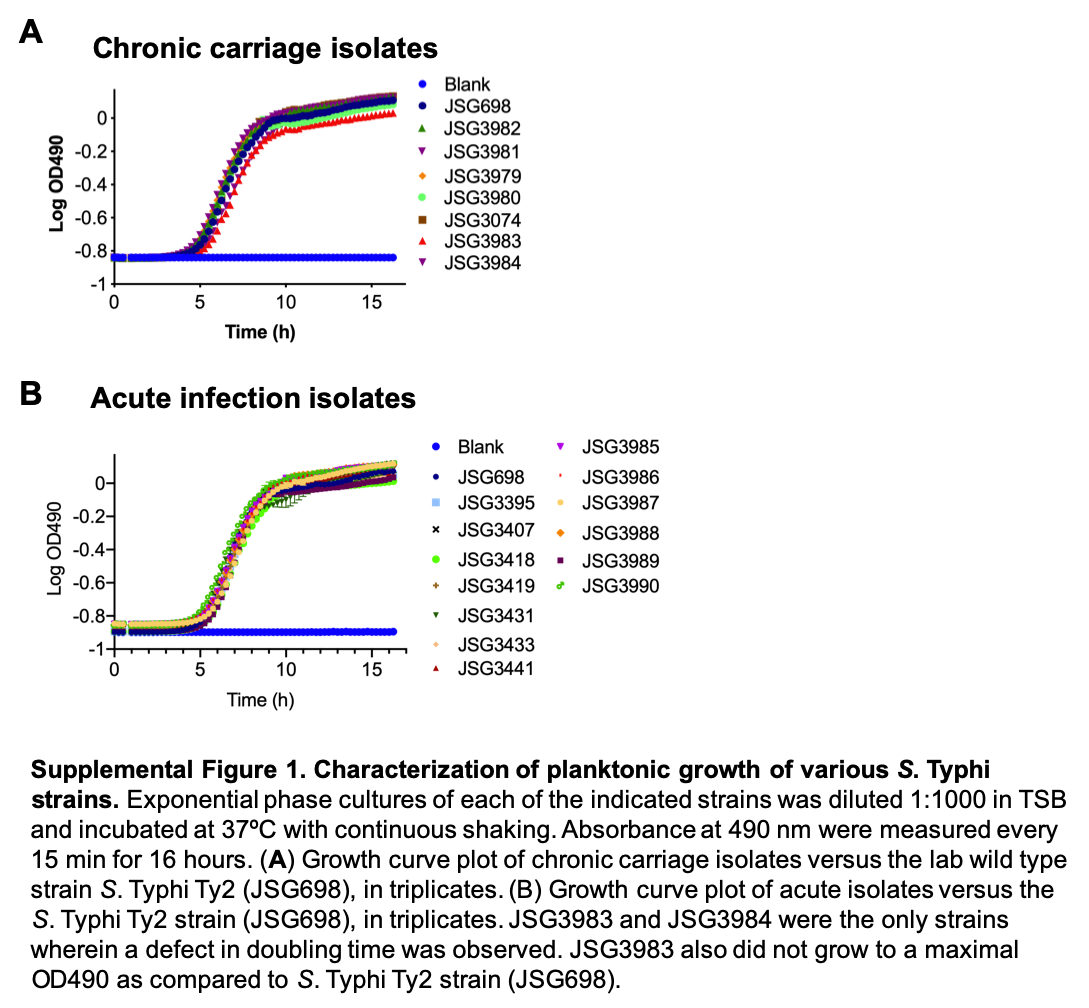

Supplement: S1 Fig — Exponential phase cultures of each of the indicated strains was diluted 1:1000 in TSB and incubated at 37°C with continuous shaking. Absorbance at 490 nm were measured every 15 min for 16 hours. (A) Growth curve plot of chronic carriage isolates versus the lab wild type strain S. Typhi Ty2 (JSG698), in triplicates. (B) Growth curve plot of acute isolates versus the S. Typhi Ty2 strain (JSG698), in triplicate. JSG3983 and JSG3984 were the only strains wherein a defect growth rate was observed. JSG3983 also did not grow to the same extent as S. Typhi Ty2 strain (JSG698). (TIF) [file ppat.1009209.s001.tif]

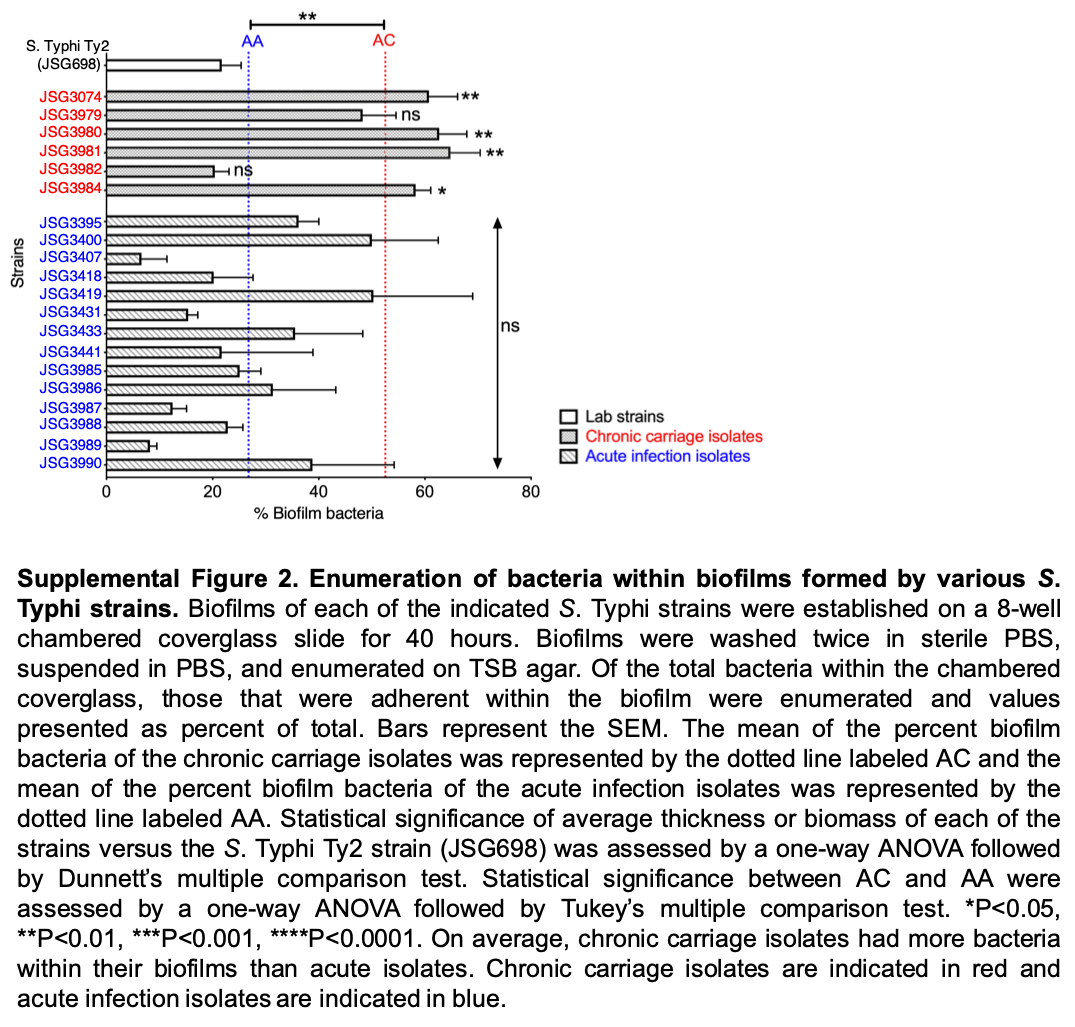

Supplement: S2 Fig — Biofilms of each of the indicated S. Typhi strains were established on a 8-well chambered coverglass slide for 40 hours. Biofilms were washed twice in sterile PBS, suspended in PBS, and enumerated on TSB agar. Of the total bacteria within the chambered coverglass, those that were adherent within the biofilm were enumerated and values presented as percent of total. Bars represent the SEM. The mean of the percent biofilm bacteria of the chronic carriage isolates was represented by the dotted line labeled AC and the mean of the percent biofilm bacteria of the acute infection isolates was represented by the dotted line labeled AA. Statistical significance of average thickness or biomass of each of the strains versus the S. Typhi Ty2 strain, JSG698 was assessed by a one-way ANOVA followed by Dunnett’s multiple comparison test. Statistical significance between AC and AA were assessed by a one-way ANOVA followed by Tukey’s multiple comparison test. *P<0.05, **P<0.01, ***P<0.001, ****P<0.0001. On average, chronic carriage isolates had more bacteria within their biofilms than acute isolates. Chronic carriage isolates are indicated in red and acute infection isolates are indicated in blue. (TIF) [file ppat.1009209.s002.tif]

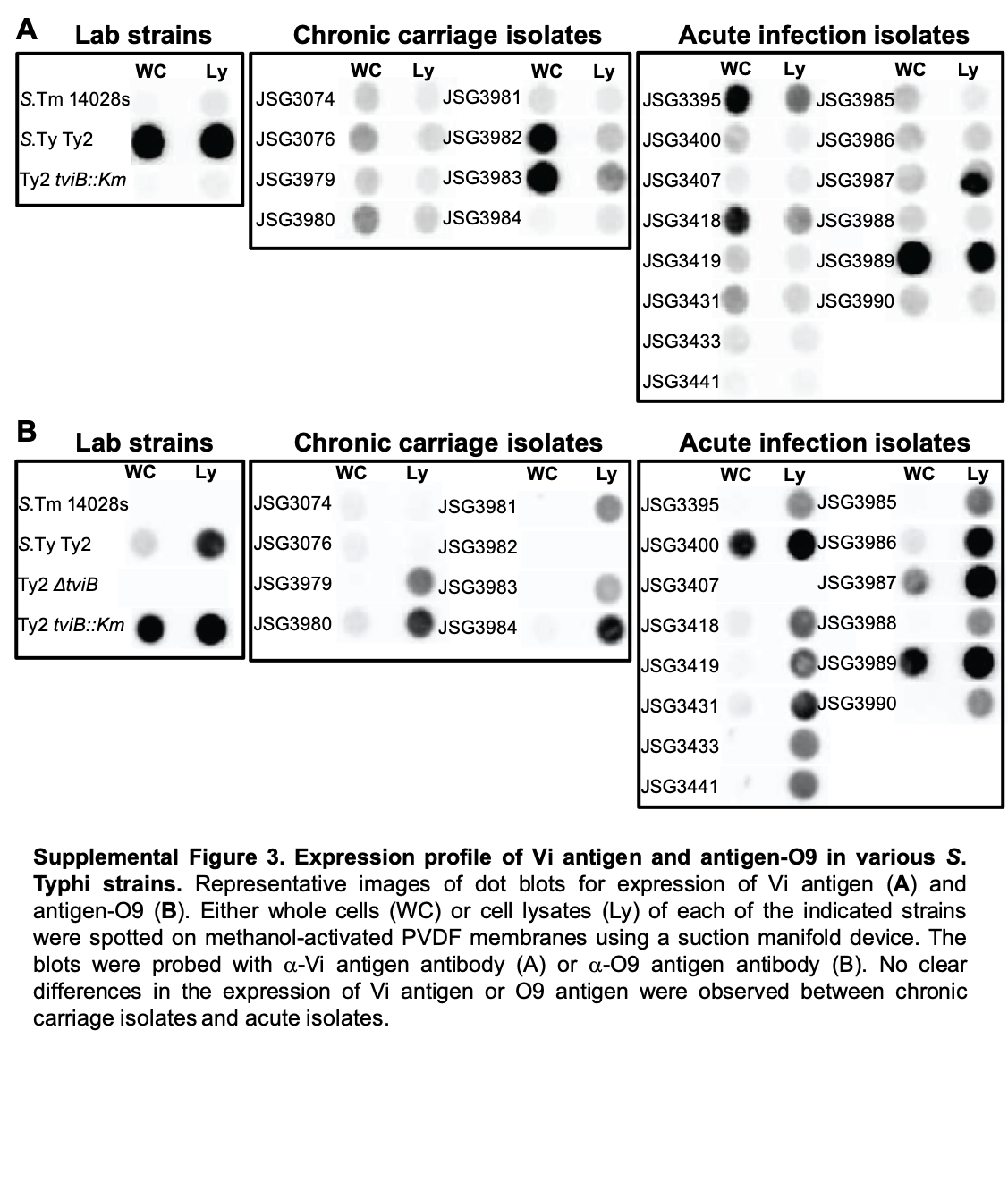

Supplement: S3 Fig — Representative images of dot blots for expression of Vi antigen (A) and LPS (B). Either whole cells (WC) or cell lysates (Ly) of each of the indicated strains were spotted on methanol-activated PVDF membranes using a suction manifold device. The blots were probed with α-Vi antigen antibody (A) or α- O9 antigen antibody (B). No clear differences in the expression of Vi antigen or O9 antigen were observed between chronic carriage isolates and acute isolates. (TIF) [file ppat.1009209.s003.tif]

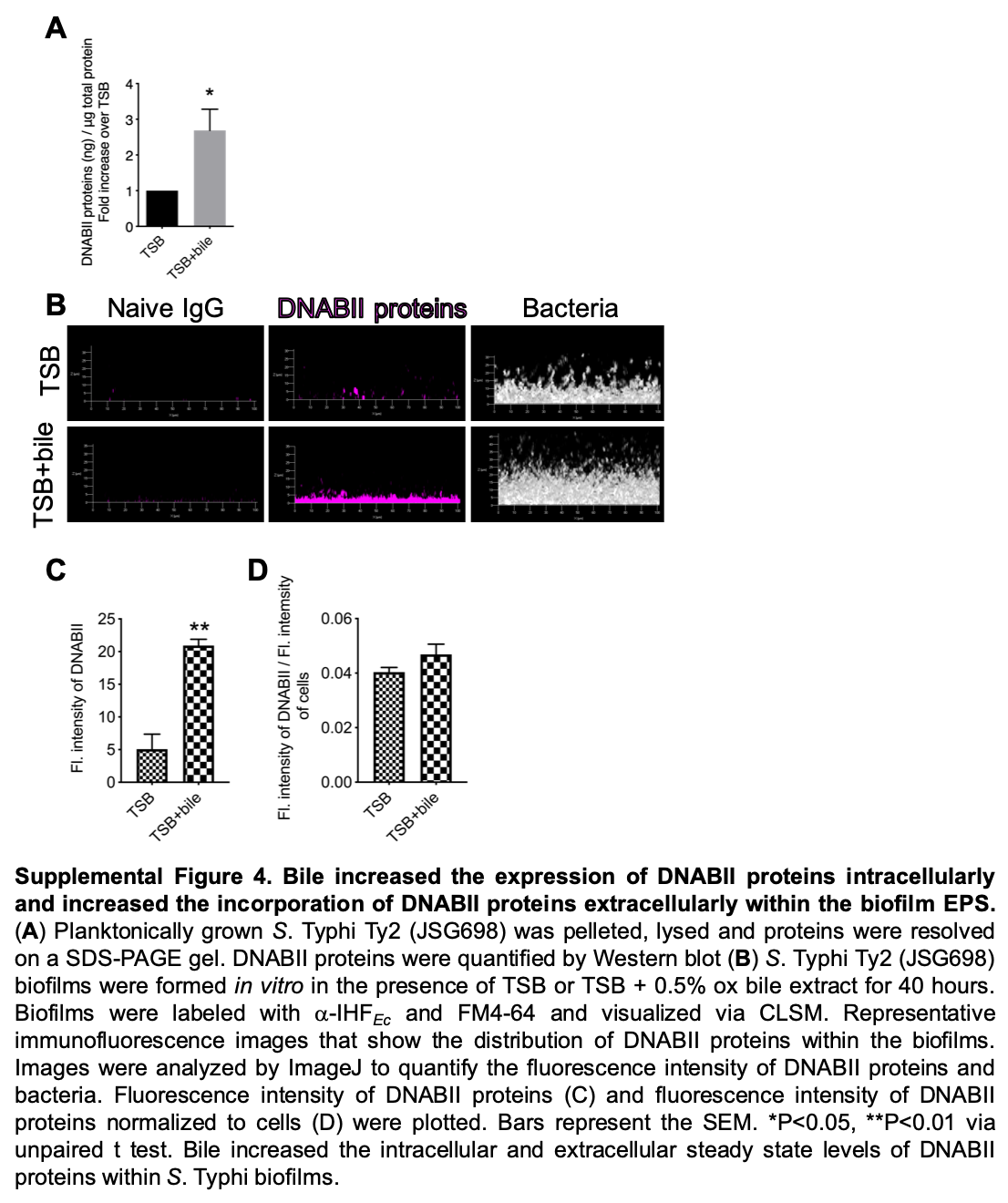

Supplement: S4 Fig — (A) Planktonically grown S. Typhi Ty2 (JSG698) was pelleted, lysed and proteins were resolved on an SDS-PAGE gel. DNABII proteins were quantified by Western blot. (B) S. Typhi Ty2 (JSG698) biofilms were formed in vitro in the presence of TSB or TSB + 0.5% ox bile extract for 40 hours. Biofilms were labeled with α-IHFEc and FM 4–64 and visualized via CLSM. Representative immunofluorescence images that show the distribution of DNABII proteins within the biofilms. Images were analyzed by ImageJ to quantify the fluorescence intensity of DNABII proteins and bacteria. Fluorescence intensity of DNABII proteins (C) and fluorescence intensity of DNABII proteins normalized to cells (D) were plotted. Bars represent the SEM. *P<0.05, **P<0.01 via unpaired t test. Bile increased the intracellular and extracellular steady state levels of DNABII proteins within S. Typhi biofilms. (TIF) [file ppat.1009209.s004.tif]
