# Supplementary material for: Enhanced biofilm and extracellular matrix production by chronic carriage versus acute isolates of Salmonella Typhi
Source: PLoS Pathog. 2021 Jan 19;17(1):e1009209. doi: 10.1371/journal.ppat.1009209 (PMC7815147; doi:10.1371/journal.ppat.1009209)
Supplement: Attachment — Submitted filename: Cover letter_revision_final_112420.docx [file ppat.1009209.s005.docx]

November 24, 2020

Dear Dr. Baumler,

We would like to thank you for considering the revision of our manuscript (MS# PPATHOGENS-D-20-02247) entitled “*Enhanced Biofilm and Extracellular Matrix Production by Chronic Carriage versus Acute isolates of Salmonella Typhi*” for publication in PLoS Pathogens. Herein, we characterized multiple *S*. Typhi strains isolated from acute infection and chronic carrier patients and identified distinct phenotypic differences between the strains based on the infection status (acute vs. chronic) of the patient. Overall, we demonstrated that during carriage, chronic *S*. Typhi isolates may undergo pathoadaptation in the gallbladder to enhance the biofilm state. Indeed, we also demonstrated that a specific antibody that targets DNABII proteins significantly disrupted biofilms formed by a chronic carrier strain, which thereby supports development for therapeutic use of this antibody to potentially treat the chronic carriage of *S*. Typhi.

In light of the excellent comments and suggestions made by the reviewers, we have shortened the introduction and discussion by 26% and 13% respectively, replotted the growth curves as semi-log plots and calculated doubling times for each of the *S*. Typhi strains. In summary, we have carefully read the comments and suggestions raised by the reviewers, addressed each (in blue) and revised the manuscript accordingly. We feel that the manuscript is now significantly improved and are thereby hopeful that these changes warrant a decision in favor of publication.

We look forward to hearing from you.

Sincerely,

Steven D. Goodman Ph.D*.*
Professor of Pediatrics
Center for Microbial Pathogenesis
Abigail Wexner Research Institute at Nationwide Children's Hospital
The Ohio State University College of Medicine
700 Children's Drive, W492
Columbus, OH 43205
(614) 355-2761

**Part I - Summary**
Reviewer #1: Devaraj et al. characterize a series of acute and chronic Salmonella Typhi strains, showing that, independent of lineage, the chronic strains tend to make “better” biofilms, consistent with their ability or selection to colonize gall stones. The experiments are generally straight forward, although the results are correlative without any molecular explanation. The paper is more wordy than necessary and could be shortened significantly. My other comments also focus on presentation.

Reviewer #2: In this study, Devaraj et al characterize a small collection of S. Typhi isolates to investigate whether strains isolated from chronic or acute typhoid fever exhibit genomic or phenotypic differences that could point towards pathoadaptation. Based on the genome sequence of these isolates, the authors generate a phylogenetic tree; this analysis fails to identify obvious patterns that correlate with disease state (chronic vs acute). The authors then analyze biofilm formation of each strain in vitro. Strains isolated from chronically-infected patients tended to form thicker biofilm and these biofilms contained more extracellular DNA and DNABII proteins. In contrast, no difference in the production of the Vi antigen were noted. Antibodies directed against S. Typhi DNABII proteins disrupted the biofilm and decreased biofilm-associated biomass.

Overall, the experiments are well-executed, the manuscript is well-written and easy to follow. All major conclusions are supported by the data. The finding that S. Typhi may adapt during chronic infections to produce more biofilm and thus ensure better gall bladder colonization is novel and exciting. Previous studies have reported effects of anti-DNABII on bacterial biofilms and as such, this concept and technology is not entirely novel. However, this was not known for S. Typhi and is new information. I only have some minor comments regarding the writing and data presentation.

**Part II – Major Issues: Key Experiments Required for Acceptance**
Please use this section to detail the key new experiments or modifications of existing experiments that should be absolutely required to validate study conclusions.

Generally, there should be no more than 3 such required experiments or major modifications for a "Major Revision" recommendation. If more than 3 experiments are necessary to validate the study conclusions, then you are encouraged to recommend "Reject".
Reviewer #1: None

Reviewer #2: None

**Part III – Minor Issues: Editorial and Data Presentation Modifications**
Please use this section for editorial suggestions as well as relatively minor modifications of existing data that would enhance clarity.
**Reviewer #1**:

1. Define DNABII proteins in the abstract
   DNABII proteins have been described in the abstract. Please refer to lines 49 – 51 in the revised manuscript.

2. Both the introduction and discussion could be shortened significantly without loss of important content.
The introduction and discussion were shortened by 26% and 13% respectively as suggested. Please refer to the revised manuscript.

3. It is not clear that the pulse-field gel experiment adds anything to the paper given the subsequent genome sequencing.

We agree with the reviewer and have removed all text and data related to the pulse-field gel experiment. Please refer to the revised manuscript.
4. Line 198. It would be better to call and label JS698 Ty2. You do this in some figures, but not all. JSG4383 isn’t in the strain table so I can’t see the genotype. I wonder if (line 200) you mean that you repaired or replaced the mutant rpoS allele with wt rather than “complemented” it in trans.

We thank the reviewer for bringing this to our attention. We have revised all instances of JSG698 to *S*. Typhi Ty2 strain (JSG698). Also, we have revised the description for strain JSG4383 to *S*. Typhi Ty2 strain *rpoS^+^* and added the strain information in **Table. 1**. Please refer to lines 172 – 174 in the revised manuscript.

5. Line 233. You don’t really mean that “LPS was absent” versus lack of antigen. Rephrase. Indeed, what do you mean by anti-LPS antibody? If it is anti-O9, for example, you just say so.

We have revised all instances of α-LPS antibody to α-O9 antigen. Please refer to lines 202 – 212 in the revised manuscript.

6. Line 244. “…also the translated proteins of IHF and HU…” is somewhat awkward.
We deleted “the translated proteins of” and now the line reads as “(recognizes both IHF and HU isolated from a large crossection of bacterial species; also the primary amino acid sequences of IHF and HU from *E. coli* and *Salmonella* are identical)”. Please refer to lines 218 in the revised manuscript.

7. Fig 1. Barely visible in Fig 1A, you demarcate acute and chronic strains with very small red and blue dots and this color scheme is carried through to 2B. You need to make this distinction more obvious by repeating the mark in 2B (larger dots would be nice) or saying it in the legend. …and why not carry it through to 2C?

We have changed the colors of axis labels for chronic carriage isolates and acute infection isolates in Figs. 2, 3 and supplementary Fig. 3 to red and blue respectively to more readily demarcate these strains. We have also indicated this difference in the respective figure legends. Please refer to revised Figs. 2, 3 and supplementary Fig. 2.

8. Supp Fig 2. Growths curves need to be plotted with the OD on a log scale.

The growth curves were revised and are now plotted on a log scale. Please refer to revised Supplementary Fig. 1.

**Minor comments**:

9. Line 58 individuals

The spelling error was corrected. Please refer to line 58 in the revised manuscript.

10. Line 60. …chronic carrier state, although not genotypically distinct…
The spelling error was corrected. Please refer to line 63 in the revised manuscript.

11. Line 119. …immune effectors, and antibiotics…
The error was corrected. Please refer to line 112 in the revised manuscript.

12. Line 121. …that negatively affect its…
This line in the original manuscript “We have recently shown that *Salmonella* undergoes pathoadaptive alterations in the gallbladder that negatively affects its virulence capabilities, yet enhances its ability to persist (19)” was deleted when the introduction was shortened. Please refer to the revised manuscript.

13. Line 124. “…that alter the transcriptional landscape of chronic carriage of Salmonella…” This doesn’t make sense as written.

When the introduction was shortened, this line was deleted. Please refer to the Introduction in the revised manuscript.

**Reviewer #2**: Fig. S2: Bacterial growth curve are easier to interpret when plotted on semi-log plot (log of OD, time in linear scale). The exponential growth phase is then clearly identifiable as a linear feature on this type of plot. The doubling time, based on exponential growth, should be calculated and reported. Also, is the growth defect referring to a slower growth rate during exponential phase or a lower recovery (density) in stationary phase? Based on the current plot, it certainly is the latter, but the former is difficult to assess.
We thank the reviewer for this insightful suggestion. The growth curves were revised and are now plotted on a log scale. Please refer to revised Supplementary Fig. 2. We calculated the doubling time for each of the *S*. Typhi strains and reported these data in a new Table. 2. Please refer to lines 165 – 168 in the revised manuscript.

I recommend that standard bacterial genetics nomenclature should be followed throughout the manuscript. It is a bit unusual to see that wild-type strains are referred to by a number assigned by the investigator. JSG698 and JSG210 should be denoted as Ty2 and 14028S in the manuscript (e.g. line 201 and Fig. 2). Also, if the tviB mutant JSG1213 was previously published by the Popoff group, please provide this reference (maybe PMID: 8574397?).

We have revised all instances of JSG698 to *S*. Typhi Ty2 strain (JSG698), and JSG210 to 14028s. The suggested reference was added to Table. 1 and line 206 (reference 34) in the revised manuscript.
